# Motivation and academic performance of medical students from ethnic minorities and majority: a comparative study

**DOI:** 10.1186/s12909-017-1079-9

**Published:** 2017-11-28

**Authors:** Ulviye Isik, Anouk Wouters, Marieke M. ter Wee, Gerda Croiset, Rashmi A. Kusurkar

**Affiliations:** 1 VUmc School of Medical Sciences, Research in Education, Amsterdam, the Netherlands; 2 LEARN! Research Institute for Learning and Education, Faculty of Psychology and Education, VU University Amsterdam, Amsterdam, the Netherlands; 3 VUmc School of Medical Sciences, P.O. Box 7057, 1007 MB Amsterdam, the Netherlands; 4 Department of Epidemiology & Biostatistics, VU University Medical Center Amsterdam, Amsterdam, the Netherlands

**Keywords:** Academic performance, Diversity, Ethnicity, Medical students, Motivation

## Abstract

**Background:**

Medical students from ethnic minorities underperform in knowledge and skills assessments both in pre-clinical and clinical education compared to the ethnic majority group. Motivation, which influences learning and academic performance of medical students, might play an important role in explaining these differences, but is under-investigated. This study aimed to compare two types of motivation (autonomous and controlled) of ethnic minority (Western and non-Western) and majority (Dutch) students, and their association with academic performance.

**Methods:**

In a cross-sectional study, all students of a Dutch medical school were invited to complete a survey including the Academic Self-Regulation Questionnaire, measuring autonomous and controlled motivation, in the academic year 2015–2016. Motivation was compared using Kruskal-Wallis test and performance was compared using One-Way ANOVA. Linear regression analysis was used to determine the association between motivation and performance (grade point average; GPA).

**Results:**

The response rate was 38.6% (*n* = 947). Autonomous motivation (AM) of non-Western students was higher than that of Dutch students in pre-clinical and clinical education (*p* < 0.05). Controlled motivation was higher in Western students than in Dutch students (pre-clinical education; *p* < 0.05). AM was associated with a higher GPA for Dutch (pre-clinical education; β = 0.33, *p* < 0.05) and Western students (clinical education; β = 0.57, *p* < 0.05) only.

**Conclusions:**

Our results show significant differences in the type of motivation between the ethnic majority and minority groups. The association of motivation with performance also differs between ethnic groups. We found that AM has a positive influence on GPA. Further research is needed to uncover the underlying mechanisms.

## Background

Medical students from ethnic minorities underperform both in pre-clinical and clinical years compared to the ethnic majority group [1, 2]. They score lower on knowledge and skills assessments, and have difficulties in procuring placements for post-graduate medical education [1, 3]. This is unexpected because students are admitted to medical schools through selection procedures, and students, irrespective of their ethnicity, enter with similar credentials and levels of academic performance.

Examiner bias has been investigated as a factor influencing such underperformance, but was found to explain it only partly [4]. Moreover, ethnicity specific factors like stereotype threat [5, 6], threat to feelings of belongingness [7], and factors not specific to ethnicity, like socioeconomic status [2, 8], that negatively influence the performance of ethnic minority students, also explain this underperformance only partly. A large part of the variance in underperformance is not yet explained in the literature.

Motivation, which has been found to be associated with learning and academic performance of (medical) students in particular, could play an important role in explaining differences between the performance of ethnic minority and majority students, especially since motivation is dynamic and can be influenced by factors in the learning environment [9–11].

Postgraduate residents in medical education from ethnic minorities in the Netherlands reported that they experienced ethnicity-related barriers like a lack of social networking abilities and personal assertiveness during the selection for specialty training [3]. They also indicated difficulty in succeeding in specialty training owing to stereotyped imaging. These types of barriers are likely to have a great impact on their motivation for the medical study, and hence their performance.

Self-Determination Theory (SDT), was applied as the framework in this study [11, 12].

According to SDT, different types of motivation exist along a continuum [11]. These are amotivation, extrinsic motivation, and intrinsic motivation. Amotivation indicates a lack of intention to act. Extrinsic motivation, which indicates that the motivation originates from external factors, can be distinguished into three subtypes: external regulation (behaviour to satisfy an external demand, e.g. external/parental pressure, or behaviour to obtain a reward or avoid a punishment), introjected regulation (e.g. internal pressure or feelings of guilt or shame), and identified regulation (valuing a behavioural goal as personally important). Intrinsic motivation indicates that the motivation originates from genuine interest. Intrinsically motivated students experience enjoyment and satisfaction while learning [11, 13]. Intrinsic motivation and identified regulation together form autonomous motivation (AM). Introjected regulation and external regulation together form controlled motivation (CM). Autonomous motivation is more desirable than controlled motivation because it seems to be associated with deep learning, better academic performance and less exhaustion [9, 10]. As per SDT, to feel autonomously motivated, three basic psychological needs should be fulfilled: feeling autonomy (feeling of choice in an action), feeling competence (feeling capable of reaching a goal) and feeling related (feeling like belonging to the group) [14]. Fulfillment of these needs in a learning environment can move a student from controlled motivation towards autonomous motivation. In contrast, non-fulfillment can move a student along the SDT continuum from autonomous motivation towards controlled motivation [15, 16].

In a recent review we reported a lack of research on motivation of ethnic minority students in medical education (Isik U, El Tahir O, Meeter M, Heymans MW, Jansma EP, Croiset G, Kusurkar RA: Factors influencing academic motivation of ethnic minority students: a review, submitted), despite its importance for the learning and performance of students. The hypothesis of this study is that ethnic minority students experience factors in the learning environment that change their motivation to the controlled type, which in turn negatively influences their academic performance. But, nothing is known in the literature about the type of motivation of ethnic minority medical students. For investigating this, a baseline measurement of this motivation and comparison with the majority group is necessary, which was done in the present study. To explore if motivation could play a role in the underperformance of ethnic minority students, we investigated the association between motivation and performance for students from different ethnic backgrounds. The research questions for this study were:

- 1. Do autonomous and controlled motivation, and academic performance (including professional behaviour) differ between ethnic majority and minority students in pre-clinical and clinical education?
- 2. What are the associations between autonomous motivation, controlled motivation and academic performance of majority and minority students in pre-clinical and clinical education?

## Methods

### Study design

The data for this cross-sectional study have been collected in the first year of a large longitudinal study called the Student Motivation and Success (SMS) study [12]. The SMS study (made up of several sub-studies) has been set up to collect data for six years about medical students’ characteristics and academic performance in order to understand the mechanisms underlying these processes during their education better. A specific part of the data collected in the SMS study (as a sub-study) was used for the present paper.

### Setting

This study was conducted at VUmc School of Medical Sciences, Amsterdam, the Netherlands. The medical study entails three years of pre-clinical education followed by three years of clinical education. The curriculum is competence-based, student-centered and vertically integrated [17].

### Participants and procedure

At the beginning of the academic year, i.e. in September 2015, all medical students enrolled at VUmc School of Medical Sciences were invited to participate in the study (*n* = 2451). The total percentage of ethnic minority students in the school was approximately 30% at the time of this study.

All students were sent an electronic survey, using Net Questionnaire. This survey included a validated motivation questionnaire, the Academic Self-Regulation Questionnaire (SRQ-A) and questions on ethnic background [18]. Performance measures were retrieved from the student administration. Analysis was conducted on anonymized data.

Written informed consent was obtained from all participants. Participation was voluntary and had no consequences for students’ academic grades. Ethical approval for this study was obtained from Ethical Review Board of the Netherlands Association for Medical Education (NVMO-ERB, file no. 388).

### Data collection

*Academic Self-Regulation Questionnaire* (SRQ-A) is based on SDT and measures individual differences in the four types of motivation or regulation (i.e., external regulation, introjected regulation, identified regulation, intrinsic motivation) for school [10]. A Dutch version of this 16-item questionnaire, adapted from the SRQ-A by Ryan and Connell [19], was used in this study [19]. Each item was scored on a 5-point Likert scale (1 *= completely not important,* 5 *= very important)*. Autonomous motivation score was calculated as an average of intrinsic motivation and identified regulation scores. Controlled motivation was calculated as an average of introjected and external regulation scores. Both validity and reliability of the Dutch version of this questionnaire have been reported in earlier studies [18, 20, 21]. The previously reported Cronbach’s alphas for the autonomous and controlled motivation subscales vary from 0.63 to 0.88 and from 0.62 to 0.84, respectively [18, 20, 21].

*Ethnic background questions* pertained to the country of birth of the student and both parents, and the language spoken with parents [2]. Ethnic minority was defined in alignment with the Statistics Bureau of the Netherlands (CBS, https://www.cbs.nl/) as “a person with at least one parent born outside the Netherlands”. Ethnic minority students were classified into the following five ethnic minority groups: ‘Turkish/Moroccan/African’, ‘Surinamese/Antillean’, ‘Asian’ (including Chinese), ‘Western’ (including European, North American and Oceanian, Indonesian, and Japanese), and ‘Other’ [2]. Students from Indonesia are categorized as ‘Western’, because of their socioeconomic and social-cultural position (https://www.cbs.nl/). Their families are mainly people born in former Dutch-India. This is the standard comparison between ethnicity groups used in the Netherlands. As these five groups were small, we combined them and thus created three final groups on the basis of ethnicity: ‘Dutch’ (majority group), ‘Western’ (minority group; including European, North American and Oceanian, Indonesian, and Japanese), and ‘non-Western’ (minority group; including ‘Turkish/Moroccan/African’, ‘Surinamese/Antillean’, ‘Asian’ and ‘Other’).

*Academic performance* was measured with the following items: Grade point average (GPA) at the first attempt on knowledge tests (on a scale of 1–10, in which 1 = poor and 10 = excellent), scores on objective structured clinical examinations (OSCEs; on a scale of 1–10), language skills test (assessed as unsatisfactory, satisfactory or good), clerkship performance grades (on a scale of 1–10), and professional behaviour judgements (assessed as unsatisfactory or satisfactory). We have taken one clerkship grade (from the clerkship that provided us with the maximal number of grades for the participating students) for each year of the study. In the first year of the clinical education two clerkship grades were considered separately: clerkship knowledge test and clerkship performance grade.

In the third year of the clinical education two clerkship grades were considered: the research internship grade and the junior doctor clerkship grade. In the junior doctor clerkship students are given direct responsibility for patients and they are expected to perform almost like graduated medical doctors.

*Confounders:* age, gender, being a first generation student, and having a medical doctor as a parent were investigated as confounders in the models and were included in the final model if they had a significant influence on the relationship between motivation and GPA. We included these variables as confounders because earlier research has shown the association of these variables with motivation and performance or hypothesized that these variables might explain the differences found in the relation between motivation and performance [2, 12, 22, 23].

### Statistical analysis

Data were screened for missing values and normality of variables.

Associations between ethnicity and the categorical variables (gender, urban background, living situation, first generation student, having a medical doctor as a parent, and the language spoken with parents) were analyzed using Chi-square tests. An One-Way ANOVA was used to check if there were age differences between the different ethnic groups and their academic performance scores. A Bonferroni adjustment was applied for the One-Way ANOVA. In these analyses, the three groups were compared, and if a significant difference was found, we conducted further follow-up analysis to find out which groups was statistically different from the other.

All analyses were conducted for students from pre-clinical and clinical education separately because the types of skills assessed in these two phases of education are different. Within this, performance on professional behaviour assessments was analysed for pre-clinical and clinical students together. We did this because professional behaviour is assessed in a longitudinal fashion throughout the curriculum and the percentage of students who received an unsatisfactory behaviour judgement was too low to allow analysis as two separate groups [24, 25]. To answer the first research question, the AM and CM (continuous variables) of non-Western, Western and Dutch students were compared using Kruskal-Wallis test. When we obtained a statistically significant difference with the Kruskal-Wallis test, Mann-Whitney U test was performed as follow-up to compare two groups at a time. The effect size was calculated using Hedges’ g because of the difference in the sample sizes of the ethnic groups.

To answer the second research question, linear regression analysis was used to study the association between motivation and academic performance (GPA). Multiple regression analysis was conducted for each ethnic group separately with the dependent variable GPA and independent variable autonomous and controlled motivation while controlling for age, gender, being a first generation student, and having a medical doctor as a parent.

In addition, students of different ethnicity groups were clustered into motivation profiles using K-means clustering on the Z-scores of their AM and CM. This analysis was performed to find explanations for the results and to understand the study population and the combination of autonomous and controlled motivation in each ethnic group. For more details on the cluster analysis method please refer to Kusurkar et al. (2013). All analyses were performed using IBM SPSS Statistic 20.0.

## Results

### Student characteristics

A total of 947 out of 2451 students participated in the SMS study; response rate 38.6%. Finally, 873 students were included in this study (see Table 1). Reasons for the excluded students were: the given student number was not registered as student (*n* = 72), and given information about study phase did not match with other data (*n* = 1), and student could not be categorized into one ethnic group (*n* = 1). The distribution of gender was 25% male (*n* = 218) and 75% female (*n* = 655), which is representative for Dutch Medical Schools [17]. Five hundred and seven (58.1%) students were categorized as ‘Dutch majority’, 55 (6.3%) as ‘Western minority’, 94 (10.8%) as ‘non-Western minority’, and 217 (24.9%) as ‘Ethnicity missing’. Non-Western minority students were more often first-generation university students compared to Dutch majority students (*p* < 0.01). Further, Dutch majority students were more often first-generation university students compared to Western minority students (*p* < 0.001). Non-Western students more often lived with their parents (*p* < 0.001) and had also more often an urban background compared to the Dutch students (*p* < 0.001). Non-Western minority students more often communicated with two or more languages with their parents compared to Western students (*p* < 0.05).

**Table 1:** Characteristics of medical students

	Dutch majority		Western minority		non-Western minority		Ethnicity missing	
	*n*	%	*n*	%	*n*	%	*n*	%
Students	507	58.1	55	6.3	94	10.8	217	24.9
Gender (male)	117	23.1	13	23.6	28	29.8	60	27.6
Age:								
Pre-clinical education - Mean (SD) Clinical education - Mean (SD)	248 258	19.65 (1.84) 24.18 (2.36)	28 27	20.54 (2.17) 24.30 (2.85)	48 46	20.42 (2.99) 24.24 (3.02)	114 103	20.56 (2.67) 24.14 (2.24)
First generation university student	255	50.3	14	25.5	61	64.9	–	–
Urban background	231	45.7	31	57.4	69	73.4	–	–
Living situation (with parents)	121	23.9	11	20.4	45	47.9	–	–
Parent is a medical doctor	72	14.3	11	20	9	9.6	–	–
Language spoken with parents (two or more languages)		†	21	38.2	56	59.6	–	–

-: missing, †: not applicable, *SD* standard deviation

Since the aim of the study was to investigate differences between different ethnic groups, we decided to leave out the ethnicity missing group (*n* = 217) from all further analysis.

### Results related to research question 1

#### 1. Academic performance

##### In pre-clinical education

The ANOVA showed a significant difference between the ethnic groups for the scores on the clerkship in the first year (*p* < 0.05). Western students had higher scores on the clerkship in the first year compared to Dutch students (*p* < 0.05). The scores on the clerkship in the first year were also significantly higher for Western students compared to non-Western students (*p* < 0.05). All other academic performance scores were not significantly different between the groups.

##### In clinical education

The ANOVA showed a significant difference between the ethnic groups for the scores on the clerkship in the second year (*p* < 0.01). The scores on the clerkship in the second year were significantly higher for Western students compared to non-Western students (*p* < 0.01; Hedges’ g = 1.0). Other performance scores were not significantly different between the groups.

#### 2. Performance on professional behaviour assessment - for all students

Few students (1.9%), pre-clinical and clinical students together, obtained an unsatisfactory professional behaviour judgement; 1.8% (*n* = 9) of the Dutch students, 5.5% (*n* = 3) of Western students, and 1.1% (*n* = 1) of non-Western student had ever received one or more unsatisfactory professional behaviour judgement(s) (Table 2). Of Dutch students 0.9%, 12.8% of Western students, and 9.1% of non-Western students failed the language tests.

**Table 2:** Academic performance grades of ‘Dutch’ majority, ‘Western’ minority, and ‘non-Western minority’ students

	All students		Dutch majority		Western minority		non-Western minority	
	*n*		*n*		*n*		*n*	
Language tests (unsatisfactory)	14	2.1%	3	0.9%	5	12.8%	6	9.1%
GPA (SD)	654	6.81 (0.85)	506	6.81(0.82)	55	6.94 (0.97)	93	6.74 (0.92)
Professional behaviour (unsatisfactory)	13	2.0%	9	1.8%	3	5.5%	1	1.1%
Pre-clinical:								
GPA (SD)	325	6.96 (0.91)	249	6.97 (0.88)	28	6.94 (1.04)	48	6.93 (0.98)
OSCE (passed)	385	88.3%	223	89.6%	26	92.9%	38	79.2%
Year 1: Clerkship grade – Mean (SD)	316	7.19 (1.16)	244	7.23 (1.08)	26	6.58 (1.58)	46	7.35 (1.18)
Year 2: Clerkship grade – Mean (SD)	117	7.48 (0.79)	94	7.51 (0.79)	9	7.44 (0.88)	14	7.29 (0.83)
Clinical:								
GPA (SD)	329	6.66 (0.75)	257	6.66 (0.72)	27	6.94 (0.91)	45	6.52 (0.81)
OSCE (passed)	304	96.2%	240	96.8%	24	100%	40	90.9%
Year 1: Clerkship knowledge test – Mean (SD)	299	7.21 (0.68)	235	7.23 (0.66)	22	7.38 (0.67)	42	7.01 (0.74)
Year 1: Clerkship grade – Mean (SD)	253	7.52 (0.32)	203	7.52 (0.30)	20	7.64 (0.35)	30	7.47 (0.38)
Year 2: Clerkship grade – Mean (SD)	208	7.77 (0.39)	170	7.77 (0.37)	14	8.00 (0.25)	24	7.59 (0.48)
Year 3: Research internship grade – Mean (SD)	195	8.15 (0.87)	157	8.12 (0.90)	20	8.50 (0.69)	18	8.00 (0.71)
Year 3: Junior doctor clerkship grade – Mean (SD)	127	8.11 (0.77)	105	8.16 (0.76)	10	8.00 (0.82)	12	7.75 (0.75)

*SD* standard deviation

#### 3. Differences in motivation between the groups

The Kruskal-Wallis test showed that there were significant differences in autonomous motivation (*p* < 0.01) and controlled motivation (*p* < 0.05) between the three ethnic groups. So, a follow-up analysis was conducted using the Mann-Whitney U test to find out which group significantly differed from the other.

##### In pre-clinical education

The Cronbach’s alpha values for reliability were 0.85 for both the AM and CM. The Mann-Whitney U test showed that AM of non-Western was significantly higher than AM of Dutch (*p* < 0.05; Hedges’ g = 0.43; Table 3). No significant difference was found in AM between the Dutch and Western students, and Western and non-Western students. CM of Western students was significantly higher than CM of Dutch students (*p* < 0.05; Hedges’ g = 0.42). The difference in CM between Dutch and non-Western students, and Western and non-Western students was not significant.

**Table 3:** Means and standard deviations of autonomous and controlled motivation of students

	All students		Dutch majority		Western minority		non-Western minority	
	Pre-clinical M (SD)	Clinical M (SD)	Pre-clinical M (SD)	Clinical M (SD)	Pre-clinical M (SD)	Clinical M (SD)	Pre-clinical M (SD)	Clinical M (SD)
AM	4.35 (0.49) *n* = 321	4.22 (0.51)*n* = 326	4.32 (0.49) *n* = 247	4.19 (0.49) *n* = 253	4.34 (0.45) *n* = 27	4.24 (0.64) *n* = 27	4.53 (0.46)* *n* = 47	4.38 (0.51)* *n* = 46
CM	1.82 (0.67) *n* = 323	1.92 (0.68) *n* = 327	1.77 (0.66) *n* = 248	1.88 (0.64) *n* = 254	2.05 (0.71)* *n* = 27	2.06 (0.86) *n* = 27	1.92 (0.67) *n* = 48	2.10 (0.71) *n* = 46

*significantly higher than Dutch group *p* < 0.05, *AM* autonomous motivation, *CM* controlled motivation, *M* mean, *SD* standard deviation

##### In clinical education

The Mann-Whitney U test showed that AM of non-Western was higher than AM of Dutch students (p < 0.05; Hedges’ g = 0.39; Table 3). The difference in AM between Dutch and Western students, and Western and non-Western students was not significant. CM did not differ significantly between the three groups.

### Results related to research question 2

#### Motivation and academic performance of students

##### In pre-clinical education

The positive association between AM and GPA for Dutch students was significant (β = 0.39; *p* < 0.01), and remained significant after adjusting for age (β = 0.33; *p* < 0.05; Table 4). The CM and GPA were significantly negatively related for the Dutch students, however after adjusting for age, autonomous motivation, and being a first generation student this relation became not significant (β = −0.03; *p* > 0.05). For the Western and non-Western students these associations were not significant.

**Table 4:** Results of regression analyses: predicted influence of type of motivation on GPA for four groups

	Dutch majority		Western minority		Non-Western minority	
	Pre-clinical B (SE)	Clinical B (SE)	Pre-clinical B (SE)	Clinical B (SE)	Pre-clinical B (SE)	Clinical B (SE)
AM	0.39** (0.11) 0.33* (0.11)	0.21* (0.09) *0.17 (0.09)*	0.70 (0.45) **0.78 (0.42)**	0.57* (0.26)	−0.06 (0.32)	−0.09 (0.24)
CM	−0.17* (0.09) **−0.03** ^**§#**^ **(0.08)**	−0.06 (0.07) ***−0.04*** ^***§***^ ***(0.08)***	−0.15 (0.29)	−0.14 (0.21) −0.10^§^ (0.20)	−0.03 (0.22)	−0.07 (0.17)

*p < 0.05, **p < 0.001, AM: autonomous motivation, CM: controlled motivation, bold = adjusted for age, italics = adjusted for gender, § = adjusted for autonomous motivation # = adjusted for being a first generation student

##### In clinical education

AM was associated with higher GPA in Dutch students. However, after adjusting for gender this relation was not significant (β = 0.17; *p* > 0.05; Table 4). For Western minority students AM was significantly associated with higher GPA (β = 0.57, *p* < 0.05). For non-Western students, no significant association between AM an GPA was found. The association between CM and GPA was not significant for the three ethnic groups.

### Additional results

#### Motivational profiles of all students

Students of the different ethnicity groups were clustered into motivational profiles. This analysis was performed to find explanations for the results from the other analyses (highly autonomously motivated, less controlled motivated, small differences in performance rates between the students). Seven outliers were removed from the cluster analyses, because these analyses are sensitive to outliers. For the included students 4 clusters were found: 1) High Autonomous Moderate Controlled (HAMC), 2) High Autonomous Low Controlled (HALC), 3) Moderate Autonomous and Moderate Controlled (MAMC), and 4) Moderate Autonomous Low Controlled (MALC; see Table 5). In addition, the distribution of the students from the different ethnic groups in the 4-clusters were considered (see Table 5). The Dutch, Western, and non-Western students were mainly in the HALC profile, 34%, 31%, and 32% respectively.

**Table 5:** Means, standard deviation of motivation, and distribution of students from ethnic groups for motivational profiles

Motivational profiles (clusters)	AM Mean (SD)	CM Mean (SD)	Dutch majority n (%)	Western minority n (%)	non-Western minority n (%)
HAMC	4.68 (0.26)	2.47 (0.44)	68 (14%)	13 (25%)	27 (29%)
HALC	4.69 (0.21)	1.40 (0.29)	167 (34%)	16 (31%)	30 (32%)
MAMC	3.81 (0.29)	2.64 (0.40)	102 (21%)	11 (21%)	17 (18%)
MALC	3.99 (0.24)	1.46 (0.32)	154 (31%)	12 (23%)	19 (20%)

*AM* autonomous motivation, *CM* controlled motivation, *SD* standard deviation, *HAMC* High Autonomous Moderate Controlled, *HALC* High Autonomous Low Controlled, *MALC* Moderate Autonomous Low Controlled, *MAMC* Moderate Autonomous and Moderate Controlled

## Discussion

In the present study we found differences in the type of motivation of ethnic minority and majority students. Since we did not find that both minority student groups (Non-Western and Western) had lower autonomous motivation and higher controlled motivation as compared to the majority, our hypothesis could not be supported its entirety in this study. Non-Western students showed higher autonomous motivation than Dutch students in the pre-clinical and clinical education. Western students showed higher controlled motivation (but not lower autonomous motivation) than Dutch students both in pre-clinical and clinical education. Furthermore, significant positive association between autonomous motivation and GPA was found for the Dutch and Western group.

Our study adds new insights to the literature because, to our knowledge, this is the first study which compares the type of motivation and its relation with performance of minority students with majority students.

The academic performance grades show that students from different ethnic groups generally performed well. A systematic review and meta-analysis in the UK, and a longitudinal study in the Netherlands, previously reported that medical students from ethnic minority groups underperform compared to the ethnic majority students [1, 2]. Our findings did not confirm these findings. We actually found that, during the clerkship in the first year of the pre-clinical education, the Western minority students performed better than Dutch majority and non-Western minority students. In a longitudinal study in another Dutch medical school all minority groups achieved lower grades in the clinical education compared to the majority students [2]. Our findings also did not confirm the findings of Stegers-Jager et al. [2]. We found that only during the clerkship in the second year of the clinical education, the Western minority students performed better non-Western minority students. The academic performance scores were not significantly different between the majority and ethnic minority groups. So, our findings suggest that ethnic minority students do not underperform compared to the ethnic majority students. An explanation, considering the motivational profiles found in our sample, could be that in our study the more motivated and better performing students participated or reported their ethnicity (and therefore could be included in our analysis).

Furthermore, we found that most students were highly autonomously motivated, with scores ranging from 4.2–4.5 (on a scale of 1–5), and less controlled motivated, with scores ranging from 1.8–2.1 (on a scale of 1–5). These findings were in line with an earlier study in which medical students reported scores ranging from 4.0–4.3 and 1.8–2.2 for autonomous and controlled motivation, respectively [20]. The small differences in academic performance between the different ethnic groups could also be explained by the motivational profiles which the participants belonged to. Most students were in HALC (33%) and MALC profiles (29%). The percentage of students in the HALC profile is higher than in an earlier study by Kusurkar et al. (26.1% in HALC) [9]. We also did not find profiles with high controlled motivation in contrast to earlier studies [9, 18]. The judgements for professional behaviour of the study population can be considered good as only 1.9% of the study population had ever received one or more unsatisfactory professional behaviour judgement(s). The percentage of students at VUmc School of Medical Sciences receiving a summative unsatisfactory professional behaviour judgement increased from 0.6% in 2008–2009 to 5.7% in 2012–2013 [25]. We thus conclude that the more motivated and better performing students have probably participated in the present study.

The Western students reported higher controlled motivation than the Dutch students in the pre-clinical and clinical education. Western students more often had a doctor as a parent compared to the other ethnic groups. These students may feel more internal or external pressure to become a doctor like their parents, which might explain that they are more controlled motivated than the Dutch students. However, more research is needed to understand this finding.

In the present study, higher (autonomous) motivation was associated with better academic performance (GPA) of only Western and Dutch students. This finding differs from the results of a recent meta-analysis which reported that motivation is positively associated with academic performance of ethnic minority students (Isik U, El Tahir O, Meeter M, Heymans MW, Jansma EP, Croiset G, Kusurkar RA: Factors influencing academic motivation of ethnic minority students: a review, submitted). Another review specific to medical students in general also showed that higher motivation was associated with better academic performance [12]. Non-Western students were more autonomously motivated than the Dutch students. For these students, their performance may not be influenced by motivation directly, but through mediating factors such as study strategy and study effort [10]. Other factors like the support of the family and friends may play a role [26]. More research is needed the understand and explain the findings for the non-Western group.

### Limitations

A limitation of this study is that the response rate was low (38.6%). The response rate of ethnic minority groups (14.3% non-Western, 8.4% Western) was also low in comparison with the majority group (77.3%). In September 2015, 69.5% Dutch students, 20.9% non-Western students, and 9.6% Western students were registered as medical students at the school. Apparently, the participants from ethnic minority groups are not representative of the whole ethnic minority medical student population. Another possible limitation is a response bias, because we found high scores on autonomous motivation and low scores on controlled motivation and especially students from HALC and MALC profiles. It remains unclear how non-responders would have completed the questionnaire. Because these students did not report their ethnicity these findings do not add to our conclusions.

### Implications for education and future research

Western minority students were more controlled motivated than Dutch majority students, and non-Western students were more autonomously motivated than the Dutch majority students. Qualitative investigation into the reasons for these differences in the types of motivation could help to understand any underlying causes. These could be important, especially if factors in the learning environment stimulate CM among the non-Western minority students. If these factors are uncovered, interventions can be developed to remove such factors from the learning environment. This is important, because CM has been associated with greater exhaustion and lower well-being of students [9]. Another implication is doing qualitative research to find out what are the mediating factors between motivation and performance for ethnic minority students. It might also be important to consider whether these relations change during their education in order to find out whether the education has in impact on the differences between the groups, and how and when to intervene to support the students.

## Conclusions

In conclusion, our hypothesis that motivation has an negative impact on the academic performance is not supported by our findings. Non-Western students showed higher autonomous motivation and Western students showed higher controlled motivation than Dutch students. Results also showed that autonomous motivation was significantly associated with higher GPA for Western and Dutch students in different phases of the education. The fact that we did not found an association between the type of motivation and GPA could mean that other mediating factors play a role. The difference between the ethnic groups cannot be readily explained, so more research is needed to uncover the underlying mechanisms.

## Abbreviations

AM: Autonomous motivation
CBS: Statistics Bureau of the Netherlands
CM: Controlled motivation
GPA: grade point average
HALC: High Autonomous Low Controlled
HAMC: High Autonomous Moderate Controlled
M: Mean
MALC: Moderate Autonomous Low Controlled
MAMC: Moderate Autonomous and Moderate Controlled
OSCE: Objective structured clinical examination
SD: standard deviation
SDT: Self-Determination Theory
SMS: Student Motivation and Success
SRQ-A: Academic Self-Regulation Questionnaire

## References

[1] Woolf K, Potts HW, McManus IC (2011). Ethnicity and academic performance in UK trained doctors and medical students: systematic review and meta-analysis. BMJ.

[2] Stegers-Jager KM, Steyerberg EW, Cohen-Schotanus J, Themmen AP (2012). Ethnic disparities in undergraduate pre-clinical and clinical performance. Med Educ.

[3] Leyerzapf H, Abma TA, Steenwijk RR, Croiset G, Verdonk P (2015). Standing out and moving up: performance appraisal of cultural minority physicians. Adv Health Sci Educ.

[4] Woolf K, McManus IC, Potts HWW, Dacre J (2013). The mediators of minority ethnic underperformance in final medical school examinations. Br J Educ Psychol.

[5] Steele CM (1997). A threat in the air. How stereotypes shape intellectual identity and performance. Am Psychol.

[6] Fischer MJ (2010). A longitudinal examination of the role of stereotype threat and racial climate on college outcomes for minorities at elite institutions. Soc Psychol Educ.

[7] Mallett RK, Wagner DE, Burrow RN, Mello ZR, Worrell F, Andretta JR (2011). Do I belong? It depends when you ask. Cult Div Ethnic Min Psy.

[8] Herweijer L, Dagevos J, Gisberts M, van Praag C (2003). Voortgezet onderwijs, beroepsonderwijs en hoger onderwijs. Rapportage minderheden.

[9] Kusurkar RA, Croiset G, Galindo-Garré F, Ten Cate O (2013). Motivational profiles of medical students: association with study effort, academic performance and exhaustion. BMC Med Educ.

[10] Kusurkar RA, Ten Cate TJ, Vos CMP, Westers P, Croiset G (2013). How motivation affects academic performance: a structural equation modelling analysis. Adv Health Sci Educ.

[11] Ryan RM, Deci EL (2000). Self-determination theory and the facilitation of intrinsic motivation, social development, and well-being. Amer Psych.

[12] Kusurkar RA, Ten Cate TJ, Van Asperen M, Croiset G (2011). Motivation as an independent and a dependent variable in medical education: a review of the literature. Med Teacher.

[13] Walls TA, Little TD (2005). Relations among personal agency, motivation, and school adjustment in early adolescence. J Educ Psych..

[14] Deci EL, Ryan RM (2000). The “what” and “why” of goal pursuits: human needs and the self-determination of behavior. Psychol Inq.

[15] Kusurkar RA, Croiset G (2015). Autonomy support for autonomous motivation in medical education. Medical Education Online.

[16] Ten Cate TJ, Kusurkar RA, Williams GC (2011). How can self-determination theory assist our understanding of teaching and learning processes in medical education. AMEE guide 59. Medical Teacher.

[17] Ten Cate O (2007). Medical education in the Netherlands. Med Teacher.

[18] Vansteenkiste M, Sierens E, Soenens B, Luyckx K, Lens W (2009). Motivational profiles from a self-determination perspective: the quality of motivation matters. J Educ Psych.

[19] Ryan RM, Connell JP (1989). Perceived locus of causality and internalization: examining reasons for acting in two domains. J Pers Soc Psychol.

[20] Wouters A, Croiset G, Schripsema NR, Cohen-Schotanus J, Spaai G, Hulsman R (2017). A multi-site study on medical school selection, performance, motivation and engagement. Adv Health Sci Educ.

[21] Wouters A, Croiset G, Schripsema NR, Cohen-Schotanus J, Spaai GWG, Hulsman RL, Kusurkar RA (2017). Students’ approaches to medical school choice: relationship with students’ characteristics and motivation. IJME.

[22] Haq I, Higham J, Morris R, Dacre J (2005). Effect of ethnicity and gender on performance in undergraduate medical examinations. Med Educ.

[23] Stegers-Jager KM, Steyerberg EW, Lucieer SM, Themmen AP (2015). Ethnic and social disparities in performance on medical school selection criteria. Med Educ.

[24] Mak-van der Vossen M, Peerdeman SM, Kleinveld JH, Kusurkar RA (2013). How we designed and implemented teaching, training, and assessment of professional behaviour at VUmc School of Medical Sciences. Med Teacher.

[25] Mak–van der Vossen M, Peerdeman S, van Mook W, Croiset G, Kusurkar R (2014). Assessing professional behaviour: overcoming teachers’ reluctance to fail students. BMC Res Notes.

[26] Dennis JM, Phinney JS, Chuateco LI (2005). The role of motivation, parental support, and peer support in the academic success of ethnic minority first-generation college students. J Coll Stud Devel.

